# FXR‐mediated epigenetic regulation of GLP‐1R expression contributes to enhanced incretin effect in diabetes after RYGB

**DOI:** 10.1111/jcmm.16339

**Published:** 2021-02-21

**Authors:** Xiangchen Kong, Linxian Feng, Dan Yan, Bingfeng Li, Yanhui Yang, Xiaosong Ma

**Affiliations:** ^1^ Shenzhen University Diabetes Institute School of Medicine Shenzhen University Shenzhen China

**Keywords:** diabetes, epigenetic regulation, FXR, GLP‐1 receptor, RYGB

## Abstract

In this study, we investigated how Roux‐en‐Y gastric bypass (RYGB) enhances glucagon‐like peptide 1 (GLP‐1) response in GK rats and explored the potential link between RYGB‐stimulated BAs/FXR signalling and GLP‐1R‐linked signalling in β‐cells, a key pathway that regulates glucose‐stimulated insulin secretion (GSIS). Here we show that RYGB restores GLP‐1R expression in GK rat islets. This involves increased total BAs as well as chenodeoxycholic acid (CDCA), leading to FXR activation, increasing FXR binding to the promoter of *Glp‐1r* and enhancing occupancy of histone acetyltransferase steroid receptor coactivator‐1 (SRC1), thus increasing histone H3 acetylation at the promoter. These coordinated events bring about increased GLP‐1R expression, resulting in greater GLP‐1 response in β‐cells. Moreover, ablation of *FXR* suppressed the stimulatory effects of GLP‐1. Thus, this study unravels the crucial role of the BAs/FXR/SRC1 axis‐controlled GLP‐1R expression in β‐cells, which results in enhanced incretin effect and normalized blood glucose of GK rats after RYGB.

## 1 INTRODUCTION

Roux‐en‐Y gastric bypass (RYGB) causes remarkable improvement in glycemic control and remission of type 2 diabetes (T2D) within days after surgery, an effect happens before significant weight loss.^1^, ^2^, ^3^ A body of evidence shows enhanced incretin effect; thus, the stimulatory effect of glucagon‐like peptide 1 (GLP‐1) on glucose‐stimulated insulin secretion (GSIS), is implicated in the beneficial effect of RYGB.^1^, ^4^, ^5^, ^6^

GLP‐1, one of the incretin hormones, is secreted by the intestinal L‐cells in response to meals.^7^ GLP‐1 exerts its insulinotropic effects by binding to GLP‐1 receptor (GLP‐1R) expressed in pancreatic β‐cells. GLP‐1‐bound receptor activates a classical signalling cascade via Gs and adenylyl cyclases, thus increasing intracellular levels of cAMP and subsequently activating its effectors protein kinase A (PKA) and exchange protein directly activated by cAMP (Epac2).^8^ Activation of the two cAMP effectors leads to a series of events including increase of β‐cell electrical activity by closure of ATP‐sensitive potassium channels (K_ATP_‐channels),^9^ enhancing Ca^2+^‐influx via phosphorylation of voltage‐dependent calcium channels,^10^ mobilization of calcium release from intracellular stores^11^ and exocytotic processes.^12^ The effects mediated by PKA and Epac2 are involved in the insulinotropic action of GLP‐1.^8^, ^13^

In T2D, the incretin effect is markedly reduced.^14^ This is in line with hyperglycaemia‐induced reduction of GLP‐1R expression and impaired cAMP‐mediated effects in β‐cells.^14^, ^15^ Strikingly, however, RYGB leads to an enhanced GLP‐1 response in β‐cells and improved incretin defect in T2D patients, an effect occurs within one week post‐surgery.^4^ Notably, RYGB also increases expression of GLP‐1R in islets.^16^ However, the underlying mechanism by which this is achieved is unclear.

It has been found that bile acids (BAs) and its nuclear farnesoid X receptor (FXR) play a crucial role in improved glucose homeostasis and remission of diabetes after RYGB and sleeve gastrectomy.^17^, ^18^ FXR, a ligand‐activated transcription factor,^19^ is identified in pancreatic β‐cells.^20^, ^21^ Activation of FXR leads to enhanced^20^, ^21^ whereas FXR KO completely abolishes the stimulatory effect of BAs on GSIS.^20^ Therefore, it is crucial to understand whether/how FXR is involved in RYGB‐improved GSIS, given the important role of FXR signalling in the beneficial effect of gastric bypass surgery on glucose homeostasis.^18^ In the present study, we demonstrate that RYGB improves the expression of GLP‐1R in β‐cells of diabetic GK rats, an effect via FXR‐regulated recruitment of acetyltransferase steroid receptor coactivator‐1 (SRC1) and an increase of the acetylation of histone H3 (ACH3) at the *Glp‐1r* promoter. Importantly, our data also show that FXR plays a key role in the GLP‐1 response and thus the incretin effects in β‐cells.

## 2 MATERIALS AND METHODS

### 2.1 Animals

Male Wistar and diabetic Goto‐Kakizaki (GK) rats (8‐10 weeks.) were obtained from SLRC Laboratory Animal company (Shanghai, China). Male Sprague Dawley (SD) rats (6 weeks.) were purchased from Animal Centre of Guangdong Academy of Medical Science (Guangzhou, China). FXR knockout mice (C57Bl/6) were provided by Prof. Youfei Guan at Dalian University, China.^22^ GLP‐1R knockout mice (C57Bl/6) were kindly provided by Prof. Youmei Feng at Capital Medical University, China. These transgenic mice were bred in the Diabetes Center at Shenzhen University, China. GK rats were randomly assigned to RYGB or sham surgery. Male FXR knockout mice at 18‐20 weeks. of age with bodyweight > 25 g were selected for RYGB or sham operation. The SD rats were randomly separated into two subgroups and treated for 2 weeks. with either an intraperitoneal injection of chenodeoxycholic acid (CDCA) (20 mg/kg bodyweight/day; Sigma‐Aldrich) or saline. The animal procedures were performed in accordance with the Principles of Laboratory Animal Care and approved by the Shenzhen University Animal Care Committee.

### 2.2 Islets

The islets isolated from rats and mice were cultured as reported previously.^23^ In brief, mice were killed by cervical dislocation and pancreatic islets were isolated by collagenase P digestion (Roche Molecular Biochemicals, Indianapolis, IN, USA) that was dissolved to a concentration of 1 mg/mL in Hank's balanced salts solution and transfused into the pancreatic ducts via the common bile duct. The islets were handpicked under a stereomicroscope and were cultured in DMEM media containing 5.5 mmol/L glucose at 37℃ and 5% CO_2_ for 24 hours prior to secretion assay.

### 2.3 Cell lines

Rat INS‐1 832/13 cells were cultured as described previously.^23^ To overexpress FXR, INS‐1 832/13 cells were retrovirally transduced with either pMX‐puro or pMX‐puro‐FXR plasmid.^24^ FXR or SRC1 knockdown INS‐1832/13 cells were generated via lentivirally transduced with either scramble or shRNA targeted against FXR mRNA or shRNA targeted against SRC1 mRNA, followed by selection with 2 μg/mL puromycin for 1 week.^24^

### 2.4 RYGB and sham surgery

The RYGB procedures on rats and mice were performed as described previously.^24^ Animals were subjected to overnight fasting and continuous isoflurane anaesthesia. For the GK‐RYGB rats, the abdomen was opened by midline incision. The stomach was divided into two by suture along the white line between the forestomach and glandular stomach. A biliopancreatic limb extending 16 cm from the ligament of Treitz was transected. The distal segment was anastomosed to the gastric remnant, and the proximal segment was drained into 30 cm of the Roux limb by side‐to‐side anastomosis. For the GK‐sham‐operated animals, the abdomen was opened through a midline incision and the viscera were gently manipulated followed by abdominal closure.

For FXR mice, the stomach was ligated between the glandular portion and the gastric fundus (forestomach). The jejunum was transected at 4 cm from the ligament of Treitz and 6 cm from the site of gastroenterostomy. The distal segment of jejunum was anastomosed to the forestomach. The sham procedure involved mobilization of the forestomach and proximal and distal jejunum and ileum without any transection.

### 2.5 Oral glucose tolerance test (OGTT)

The Wistar and GK rats were fasted overnight. Blood glucose was measured at 0, 15, 30, 60 and 120 minutes after administration of 1 g/kg glucose through oral gavage, as described previously.^24^

### 2.6 Measurement of plasma bile acids

The levels of total bile acids and chenodeoxycholic acid (CDCA) were determined by using the total bile acid test kit (Sigma‐Aldrich, St. Louis, MO, USA) and CDCA ELISA kits (Cell Biolabs, San Diego, CA, USA) in accordance with the instructions.

### 2.7 Western blotting analysis

Total protein was extracted from INS‐1 832/13 cells or islets and immunoblotted as described previously.^23^ The antibodies were used: GLP‐1R (1:1000, sc‐34637 and sc‐390773, Santa Cruz, CA, USA),^25^, ^26^ GAPDH (1:3000, 5174, Cell Signaling Technology, MA, USA) and β‐actin (1:10 000, A5441, Sigma‐Aldrich, MO, USA).

### 2.8 Real‐Time PCR (qPCR) analysis

Total RNA in INS‐1 832/13 cells or islets was extracted by TRIzol reagent (Invitrogen, Carlsbad, CA, USA). One μg of RNA was used to make cDNA using PrimeScript™ RT reagent Kit (Takara, Tokyo, Japan). qPCR assay was performed on QuantStudio 5 Real‐time PCR System using SYBR Green (Promega, Madison, WI, USA). The primer sequences are listed in Table S1. GLP‐1R expression was normalized to GAPDH or β‐actin.

### 2.9 Construction of luciferase plasmid and promoter reporter assay

Rat promoter fragment of *Glp‐1r* (−1941 bp ‐ +7 bp) was amplified by PCR using the primers listed in Table S2 and was inserted into pGL3‐basic luciferase reporter vector. Mutation of the putative FXR binding site from −436 bp to −431 bp in the *Glp‐1r* promoter was performed using the primers listed in Table S2. For determination of *Glp‐1r* promoter activity, pGL3‐Glp‐1r and Renilla luciferase plasmids were transfected into 293T cells. Then, the cells were stimulated with 5 μmol/L GW4064 for 2 hours. *Glp‐1r* promoter activity was examined using the Dual‐Luciferase Reporter Assay kit (Promega, Madison, WI, USA). The Renilla luciferase plasmid was selected for normalization of luciferase activity.

### 2.10 Chromatin immunoprecipitation (ChIP) assay

ChIP assay was performed as described previously.^24^ Briefly, the lysates from 10^6^‐10^7^ formaldehyde cross‐linked cells were incubated overnight with 2 μg of control IgG or antigen‐specific antibodies, followed by incubation of protein A agarose beads for 3 hours. DNA fragments were quantified by qPCR with the primers listed in Table S3.

### 2.11 Insulin measurements

Insulin secretion of islets and INS‐1 832/13 cells was determined as described previously.^23^ Briefly, the cells were pre‐incubated in 1 mL KRB buffer for 1 hour, followed by stimulation with 16.8 mmol/L glucose or 6 mmol/L glucose in the absence or presence of 10 nmol/L GLP‐1 for 30 minutes. The insulin level was determined using the Insulin Ultrasensitive ELISA kit (ALPCO Diagnostics, Salem, NH).

### 2.12 Determination of cAMP levels

The islets or INS‐1 832/13 cells were cultured in 5.5 mmol/L glucose medium for 24 hours. Then, the cells were stimulated for 30 minutes in KRB buffer containing 16.8 mmol/L glucose with or without 10 nmol/L GLP‐1. Intracellular cAMP levels were examined using an ELISA kit according to the manufacturer's instructions.

### 2.13 Statistical analysis

All data are expressed as mean ± SEM for the indicated number of experiments (n). The SPSS Statistics 20 was used to statistical analysis. Sample groups were compared using the independent t test or one‐way ANOVA with least significant difference (LSD) post hoc test. Data were considered significant when *P* < 0.05. The SigmaPlot 10 software was used to graph.

## 3 RESULTS

### 3.1 FXR activation increases expression of GLP‐1R in β‐cells

**Figure 1:**
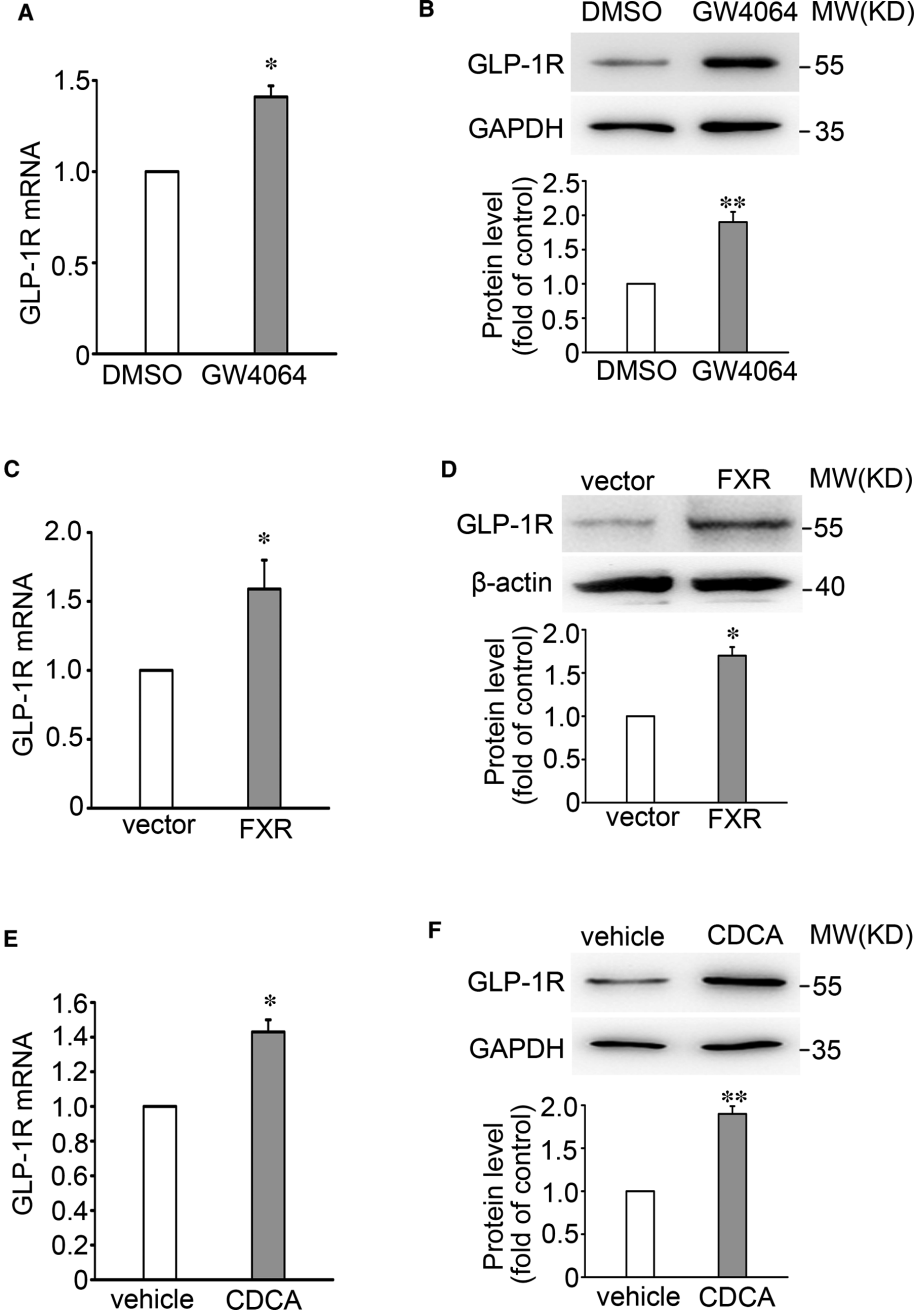
FXR agonists promote expression of GLP‐1R in β cells. A, B, GLP‐1R mRNA (A) and protein (B) were determined in INS‐1 832/13 cells by treatment with DMSO or 5 μmol/L GW4064 for 2 h (A) and 48 h (B), respectively. Bars represent means ± SEM, n = 3 (A) or 4 (B). *P < 0.05; **, *P* < 0.01. C, D, GLP‐1R mRNA (C) and protein (D) were determined in vector control and FXR overexpressing INS‐1 832/13 cells. Bars represent means ± SEM, n = 3. *P < 0.05. E, F, GLP‐1R mRNA (E) and protein (F) were examined in islets isolated from SD rats. The rats were subjected to intraperitoneal injection of CDCA at a dose of 20 mg/kg for 2 wks. Bars represent means ± SEM, n = 3 rats per group. *P < 0.05; **P < 0.01

We have reported previously that FXR knockdown (KD) suppresses the effect of GLP‐1 in β‐cells.^27^ Here we explored whether this is due to diminished expression of GLP‐1R in β‐cells. We first examined whether FXR activation affects GLP‐1R expression by treating INS‐1 832/13 cells with the FXR agonist GW4064 for 2 and 48 hours, respectively. qPCR and Western blotting results indicate that treatment with GW4064 results in a remarkable increase of GLP‐1R mRNA and protein expression (*P* < 0.01) (Figure 1A,B). The similar observations were also made in FXR overexpression INS‐1 832/13 cells (Figure 1C,D). Of note, intraperitoneal injection of SD rats with the endogenous FXR agonist CDCA for 14 days led to an obvious increase of GLP‐1R expression (Figure 1E,F), confirming the stimulatory effect of the FXR agonist on GLP‐1R expression in vivo.

**Figure 2:**
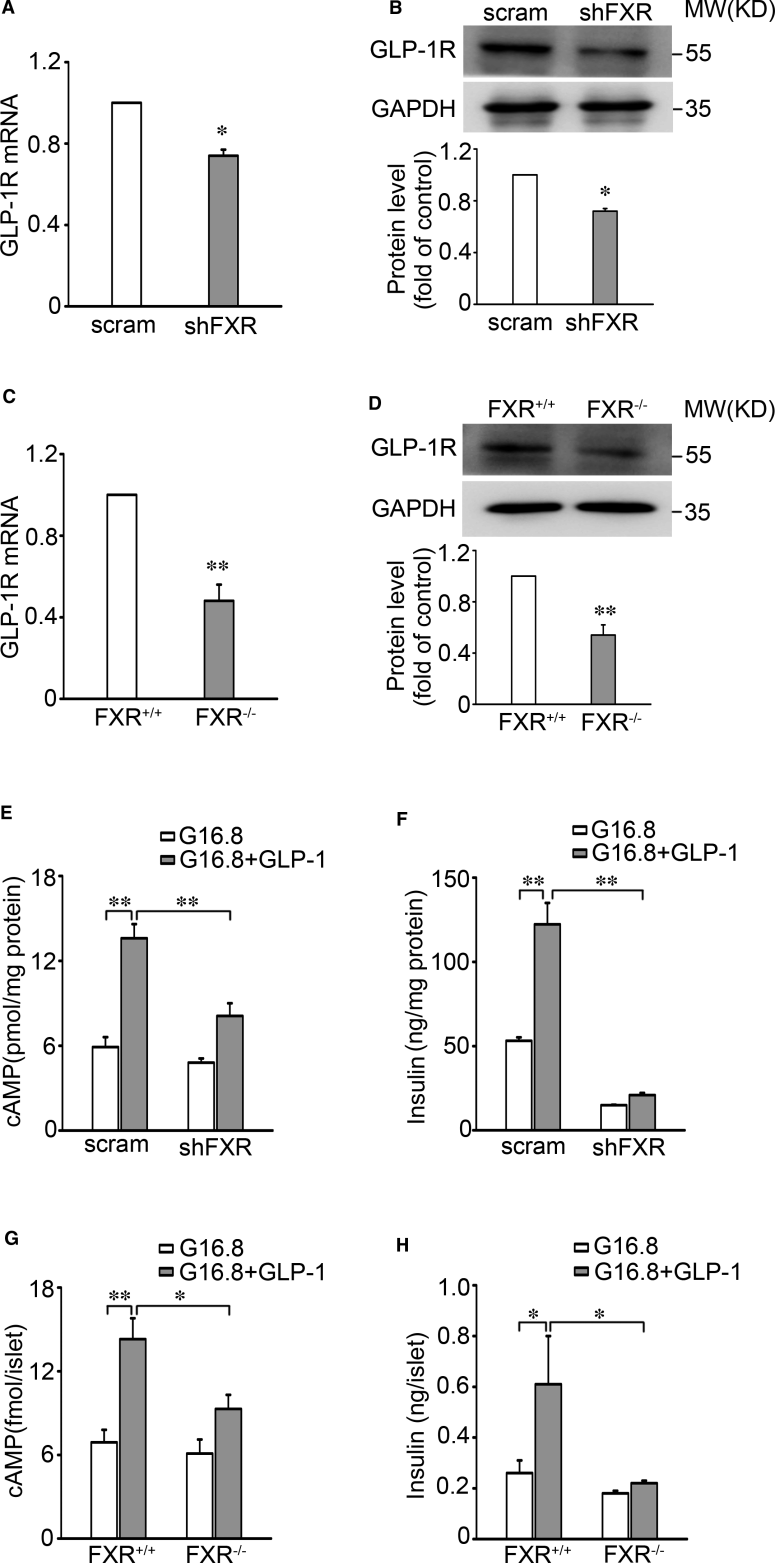
Decreased expression of GLP‐1R in islets from FXR^−/−^ mice. A, B, qPCR (A) and Western blot (B) analysis of GLP‐1R mRNA (A) and protein (B) expression in scramble control and shFXR INS‐1 832/13 cells. Bars represent means ± SEM, n = 4 (A) or 3 (B). *P < 0.05. C, D, The expression of GLP‐1R mRNA (C) and protein (D) was determined in islets from FXR^+/+^ and FXR^−/−^ mice. Bars represent means ± SEM, n = 5 (C) or 4 (D). **P < 0.01. E, F, cAMP level (E) and insulin secretion (F) were assayed in scramble and shFXR INS‐1 832/13 cells. The cells were stimulated with 16.8 mmol/L glucose in the absence or presence of 10 nmol/L GLP‐1 for 30 min. Data represent means ± SEM, n = 4. **P < 0.01. G, H, cAMP level (G) and insulin secretion (H) were determined in islets from FXR^+/+^ and FXR^−/−^ mice. Bars represent means ± SEM, n = 4 (G) or 9 (H). *P < 0.05; **P < 0.01

We next determine the role of FXR in regulation of GLP‐1R expression in β‐cells by knocking down FXR using a shRNA in INS‐1 832/13 cells and found that FXR KD led to a decreased expression of GLP‐1R (Figure 2A,B). Consistent with the observations in FXR KD INS‐1 832/13 cells, islets from FXR^−/−^ mice displayed ~52% and ~46% (*P* < 0.01) lower levels of GLP‐1R mRNA and protein, respectively, as compared with those of FXR^+/+^ controls (Figure 2C,D). Decreased level of GLP‐1R expression was paralleled by a reduced effect of GLP‐1 on cAMP generation and GSIS in FXR KD INS‐1 832/13 cells (Figure 2E,F, column 4 vs. 3) and FXR^−/−^ islets (Figure 2G,H, column 4 vs. 3). By contrast, GLP‐1 exerted pronounced stimulatory effects on cAMP level (*P* < 0.01) and GSIS (*P* < 0.01) in control INS‐1 832/13 cells (Figure 2E,F, column 2 vs. 1) and FXR^+/+^ islets (Figure 2G,H, column 2 vs. 1).

### 3.2 FXR activation promotes the occupancy of FXR at the promoter of *Glp‐1r* to increase GLP‐1R expression

**Figure 3:**
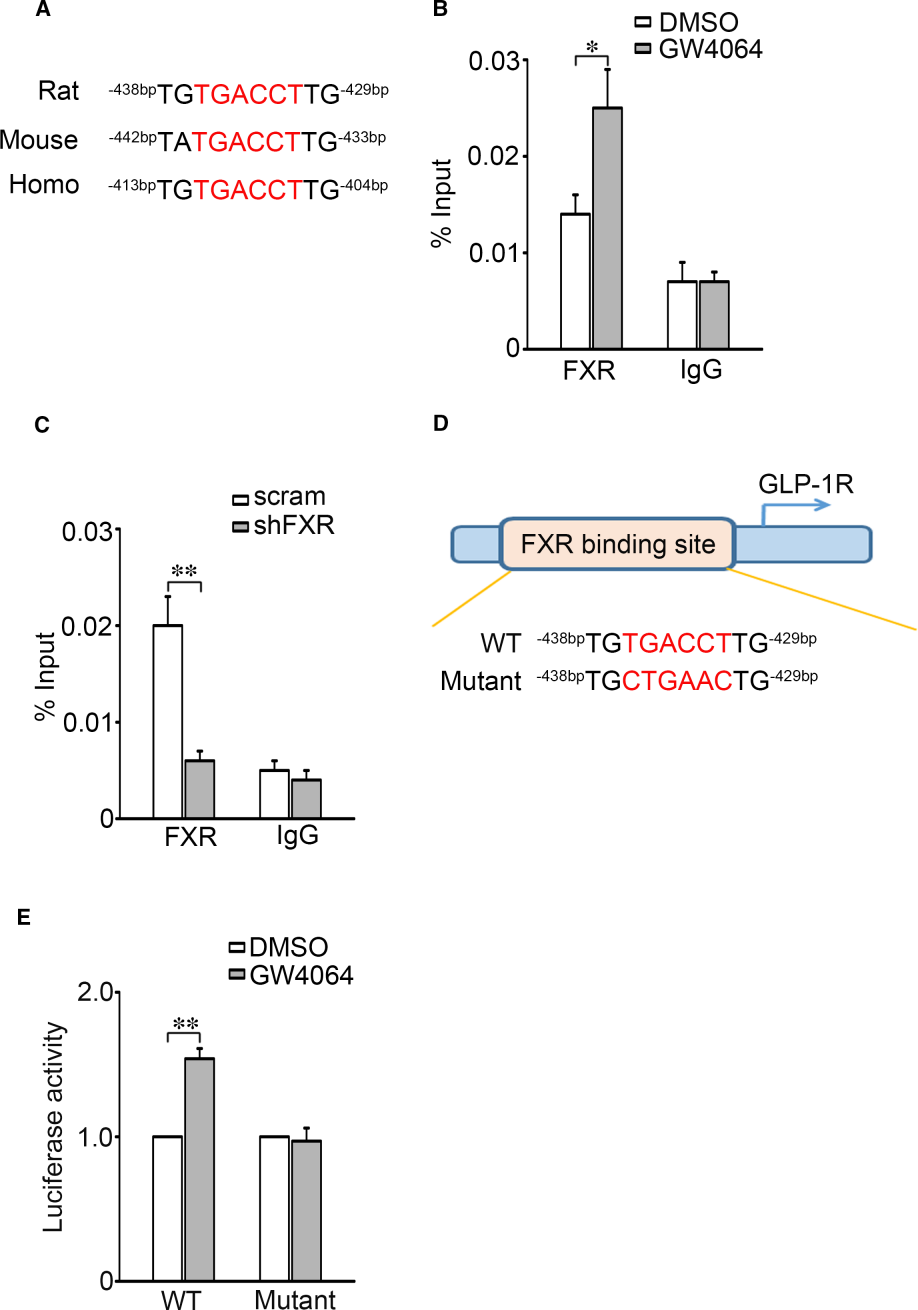
FXR activation promotes FXR binding to the promoter of GLP‐1R. A, Alignment of putative FXR binding element (FXRE) at the *Glp‐1r* promoter in various species. B, C, The occupancies of FXR at the *Glp‐1r* promoter were determined in INS‐1 832/13 cells treated with 5 μmol/L GW4064 for 2 h (B) or transfected with either *scramble* or *shFXR* (C). Control IgG was used as a negative control. *P < 0.05; **P < 0.01. D, Mutation of FXRE at the *Glp‐1r* promoter. E, Effect of GW4064 on the *Glp‐1r* promoter activity. Bars represent means ± SEM, n = 6. **P < 0.01

To clarify how FXR mediates GLP‐1R expression, we inspected the nucleotide sequence in the *Glp‐1r* locus and found a consensus ‘TGACCT’ sequence of the FXR binding site (FXRE) across different species in the *Glp‐1r* promoter (Figure 3A). ChIP assay indicated that GW4064 treatment (Figure 3B) promoted, whereas FXR KD decreased (Figure 3C) occupancy of FXR at the *Glp‐1r* promoter. To examine whether the FXRE is critical for FXR‐mediated *Glp‐1r* transcription, we constructed a luciferase reporter driven by either the wild‐type FXRE from *Glp‐1r* promoter or its mutant form ‘CTGAAC’ (Figure 3D) and examined the effect of FXR agonist GW4064 on the luciferase reporter gene activity. This revealed that treatment with GW4064 enhanced the reporter activity driven by the wild‐type, but not the mutant FXRE (Figure 3E), confirming that FXRE in the *Glp‐1r* promoter is required for FXR‐regulated GLP‐1R expression.

**Figure 4:**
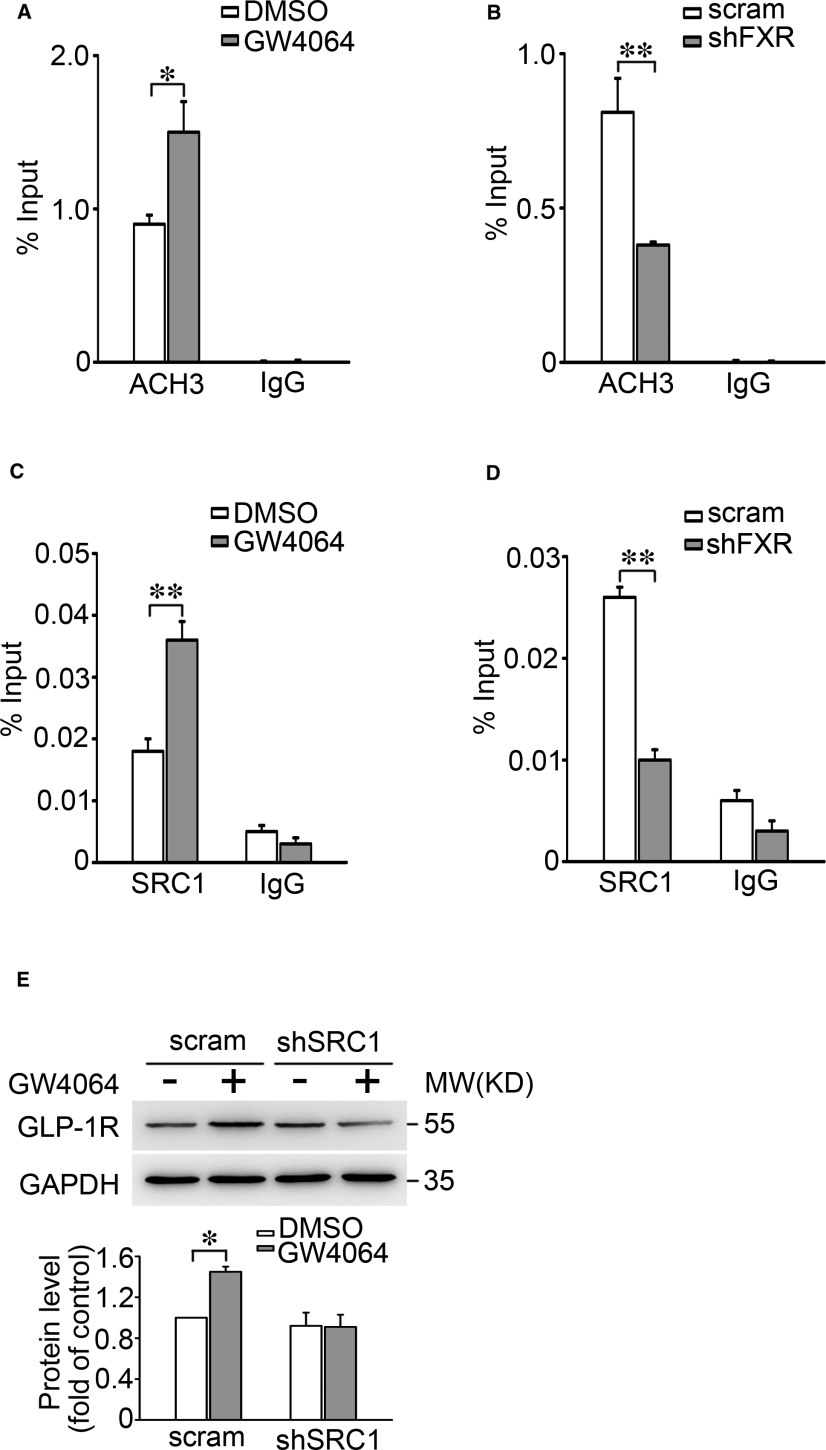
FXR mediates histone 3 acetylation, SRC1 binding to the promoter of *Glp‐1r* A, C, ChIP assays were employed to determine the histone H3 acetylation (A) and SRC1 binding (C) at the *Glp‐1r* promoter in INS‐1 832/13 cells stimulated with DMSO or 5 μmol/L GW4064 for 2 h. IgG was used as a negative control. Bars represent means ± SEM of 4 (A) or 3 (B) independent experiments. *P < 0.05. B, D, As in (A, C), but ChIP assays were performed in scramble or shFXR INS‐1 832/13 cells. Bars represent means ± SEM, n = 3. **P < 0.01. E, GLP‐1R protein expression was determined in scramble or shSRC1 INS‐1 832/13 cells stimulated with DMSO or 5 μmol/L GW4064 for 48 h. Bars represent means ± SEM, n = 3. *P < 0.05

To identify the mechanisms that FXR regulates *Glp‐1r* promoter activity, we determined the effect of FXR on ACH3 at *Glp‐1r* promoter, as ACH3 is one of the histone post‐translational modifications that promote gene transcription.^28^ The data showed that FXR activation promoted (Figure 4A), whereas FXR KD decreased (Figure 4B) ACH3 at the *Glp‐1r* promoter. Given the important role of acetyltransferase SRC1 in FXR‐mediated ACH3 at target gene promoter,^29^ we next examined whether SRC1 mediates ACH3 of *Glp‐1r* promoter in INS‐1 832/13 cells and found that treatment with GW4064 obviously enhanced SRC1 binding to the *Glp‐1r* promoter (Figure 4C). By contrast, FXR KD decreased the binding of SRC1 to the *Glp‐1r* promoter in INS‐1 832/13 cells (Figure 4D). We further explored whether SRC1 is involved in FXR‐mediated GLP‐1R gene expression. The scramble or shSRC1 INS‐1 832/13 cells were treated with either GW4064 or control DMSO. This revealed that GW4064 induced a striking increase (*P* < 0.01) of GLP‐1R expression in scramble cells, but failed to do so in SRC1 knockdown cells (Figure 4E). These results indicate that SRC1 is responsible for FXR‐controlled expression of GLP‐1R in β‐cells.

### 3.3 RYGB increases GLP‐1R expression in islets of GK rats

**Figure 5:**
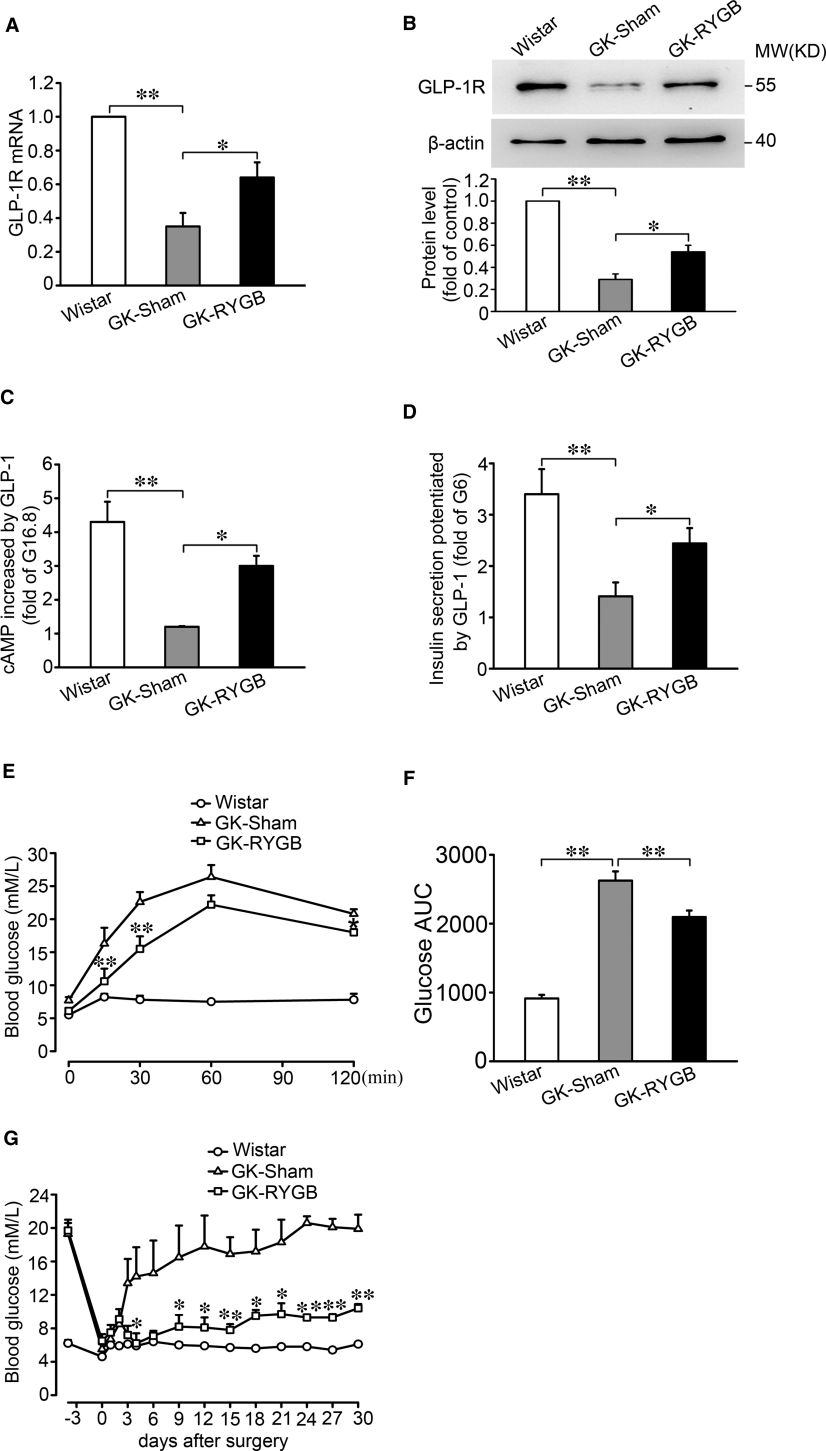
Expression of GLP‐1R in islets of GK rats after RYGB. A, B, The expression of GLP‐1R mRNA (A) and protein (B) in islets from Wistar, GK‐Sham and GK‐RYGB rats. Bars represent means ± SEM of 4 (A) and 3 (B) independent experiments. *P < 0.05; **P < 0.01. C, cAMP levels were determined in islets from Wistar, GK‐Sham and GK‐RYGB rats. The islets were stimulated with 16.8 mmol/L glucose in the absence or presence of 10 nmol/L GLP‐1 for 30 min. Values were normalized to islets and expressed as fold changes of the islets stimulated with 16.8 mmol/L glucose alone. Bars represent means ± SEM, n = 4. *P < 0.05; **P < 0.01. D, Insulin secretion was stimulated by 6 mmol/L glucose and 10 nmol/L GLP‐1 for 30 min in islets from Wistar, GK‐Sham and GK‐RYGB rats. Values were presented as fold increase at 6 mmol/L glucose. Bars represent means ± SEM, n = 4 per group. *P < 0.05; **P < 0.01. E, Glucose levels at 0, 15, 30, 60 and 120 min after administration of 2 g/kg glucose by oral gavage in Wistar, GK‐Sham and GK‐RYGB rats. Data represent means ± SEM, n = 3‐6 rats per group. **P < 0.01 vs. GK‐Sham. F, Area under the curves (AUC) was calculated for OGTT. Data are means ± SEM, n = 3‐6 rats per group, **P < 0.01. G, Non‐fasting plasma glucose was collected at 10:00 AM on the indicated days in Wistar, GK‐Sham and GK‐RYGB rats. Data represent means ± SEM, n = 3. *P < 0.05; **P < 0.01 vs. GK‐Sham

RYGB leads to increased incretin response in T2D patients.^4^, ^30^ To determine whether RYGB influences expression of GLP‐1R in islets, we performed sham and RYGB operations in diabetic GK rats and analysed GLP‐1R mRNA and protein by qPCR and Western blotting approaches. This revealed that the levels of GLP‐1R mRNA and protein in GK‐RYGB islets were ~1.82‐fold (*P* < 0.05) and ~1.86‐fold (*P* < 0.05) higher than that of GK‐sham islets, respectively (Figure 5A,B, column 3 vs. 2). Increased expression of GLP‐1R correlated with greater effects of GLP‐1 on cAMP generation and GSIS in islets from GK‐RYGB rats. Thus, treatment with 10 nmol/L GLP‐1 led to a ~2.9‐(*P* < 0.01) and ~2.4‐fold (*P* < 0.01) increase of cAMP level and GSIS, respectively, in islets from GK‐RYGB rats, the potency of GLP‐1 significantly greater than that (only ~1.2‐ and ~1.4‐fold stimulation, respectively; *P* > 0.05) in GK‐sham islets (Figure 5C,). Moreover, determination of blood glucose level by OGTT revealed that GK‐RYGB rats displayed an improved glycemic control (Figure 5E,F). Thus, GK‐RYGB rats remained the blood glucose level at ~10 mmol/L (*P* < 0.01), as opposed to that (~20 mmol/L) of GK‐sham throughout 30 days of post‐surgery (Figure 5G), consistent with remission of diabetes.

### 3.4 Failure of RYGB to increase GLP‐1R expression in islets from FXR^−/−^ mice

**Figure 6:**
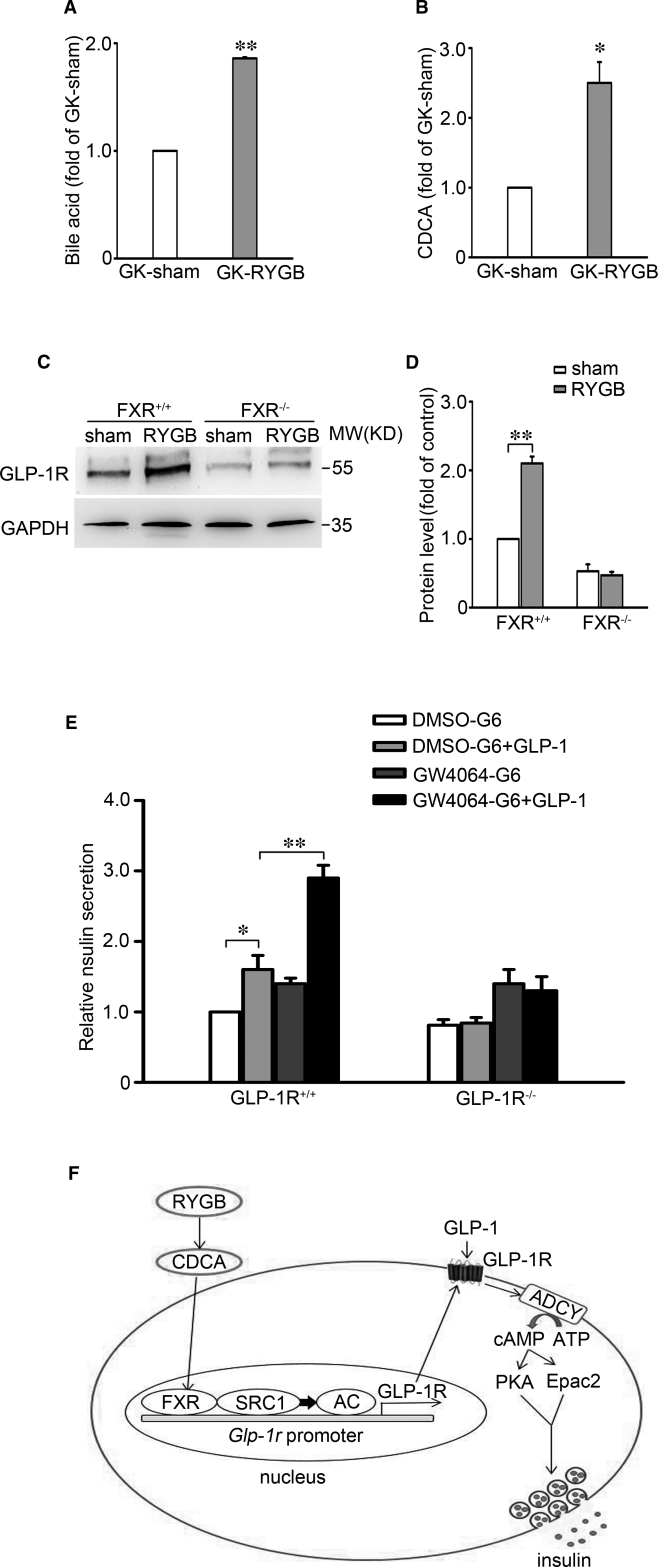
Absent of RYGB‐increased GLP‐1R expression in FXR^−/−^ mice. A, B, The levels of plasma total bile acids (A) and CDCA (B) in GK‐Sham and GK‐RYGB rats. Bars represent means ± SEM, n = 3. *P < 0.05; **P < 0.01. C, The GLP‐1R protein expression in islets from FXR^+/+^ and FXR^−/−^ mice after RYGB or sham surgery. GAPDH was used as internal control. D, Statistical analysis of the data from (C). Bars represent means ± SEM, n = 3. **P < 0.01. E, Insulin secretion was determined in islets from GLP‐1R^+/+^ and GLP‐1R^−/−^ mice. The islets were cultured in the medium containing 5 μmol/L GW4064 or DMSO for 48 h, followed by stimulation with 6 mmol/L glucose or 6 mmol/L glucose and 10 nmol/L GLP‐1 for 30 min. Bars represent means ± SEM, n = 3‐5. *P < 0.05; **P < 0.01. F, Schematic representation of the molecular mechanism of FXR/SRC1‐controlled expression of GLP‐1R in β‐cells after RYGB. See text for details

The data showed that the levels of total BAs and CDCA in GK‐RYGB rats were ~1.8‐ (*P* < 0.01) and ~2.4‐fold (*P* < 0.05) higher than that of GK‐sham rats, respectively (Figure 6A,B). This is consistent with a more pronounced FXR activation in GK‐RYGB rat β‐cells, as reported previously.^24^ We next determined the importance of FXR in RYGB‐induced expression of GLP‐1R by performing sham and RYGB operations on FXR^+/+^ and FXR^−/−^ mice and analysing GLP‐1R protein in the islets from these mice 4 wks. post‐surgeries. As shown in Figure 6C,D, GLP‐1R protein level is obviously increased in RYGB mouse islets (*P* < 0.01), as compared with the sham controls in FXR^+/+^ mice. By contrast, GLP‐1R protein remained largely constant between RYGB and sham groups in FXR^−/−^ mice, indicating that deletion of *FXR* abolishes the regulatory effect of RYGB on GLP‐1R expression. These data suggest that RYGB‐promoted GLP‐1R expression requires the participation of FXR.

Moreover, we also confirmed that FXR activation enhances the potency of GLP‐1 by experiments performed in islets from GLP‐1R^+/+^ and GLP‐1R^−/−^ mice. In these experiments, the islets were pre‐incubated in the medium containing GW4064 or DMSO for 48 hours, followed by treatment with 6 mmol/L glucose in the absence or presence of 10 nmol/L GLP‐1 for 30 minutes. This revealed that pre‐treatment with GW4064 markedly potentiated the stimulatory action of GLP‐1 (thus ~3‐fold vs. ~1.6‐fold increase in GSIS in GW4064‐ and DMSO‐treated islets, respectively; *P* < 0.01) (Figure 6E, column 4 vs. 2). Noticeably, GLP‐1R KO completely abolished GW4064‐enhanced insulinotropic effect of GLP‐1 (Figure 6E, column 8 vs.7). These results indicate that GW4064‐enhanced potency of GLP‐1 is a consequence of increased expression of GLP‐1R in β‐cells. It is also worthy of noting that GLP‐1 failed to promote GSIS in GLP‐1R KO mice (Figure 6E, column 6 vs. 5), as expected.

Collectively, this series of complementary experiments indicate that RYGB‐improved incretin effect can most likely be attributed to FXR‐regulated expression of GLP‐1R in β‐cells. Thus, RYGB leads to FXR activation, via increasing circulating total BAs and CDCA, enhancing the ability of SRC1 to bind to the promoter of *Glp‐1r* gene and subsequent stimulates GLP‐1R expression (Figure 6F). This would result in increased GLP‐1 response in the β‐cells.

## 4 DISCUSSION

In the present study, we demonstrate that GLP‐1R expression is improved in β‐cells from diabetic GK rats after RYGB, which significantly increases diabetic β‐cell responsiveness to GLP‐1, thereby enhances efficiency of GLP‐1 on GSIS and ameliorate hyperglycaemia in GK rats. For the first time, our data revealed that FXR is involved in the stimulatory effect of GLP‐1 in β‐cells. We show that FXR activation induces expression of GLP‐1R via FXR/SRC1‐mediated increase of ACH3 at the promoter of *Glp‐1r* gene. Thus, these findings suggest a key role of FXR‐regulated expression of GLP‐1R in enhanced GLP‐1 response and improved β‐cell function in diabetes after RYGB.

We demonstrate that FXR plays a critical role in the GLP‐1 response in β‐cells. This is consistent with the failure of GLP‐1 to stimulate AMP generation and GSIS in islets of FXR KO mice (Figure 2G,H) as well as in FXR KD INS‐1 832/13 cells (Figure 2E,F). Based on these findings and coupled with fact that GLP‐1 potentiates GSIS in β‐cells,^13^ it is justifiable to conclude that increased efficacy of GLP‐1 by FXR activation would account, at least partly, for enhanced GSIS observed in healthy primary β‐cells^20^ as well as the β‐cells from diabetic GK rats.^24^ Importantly, our study also demonstrates that *Glp‐1r* is one of the target genes of FXR and mediates FXR activation‐enhanced incretin effect in β‐cells. Five observations corroborate this concept. First, the FXR binding site (FXRE) exists in the *Glp‐1r* promoter (Figure 3A). Activation of FXR with GW4064 induced an increase of FXR binding to the *Glp‐1r* promoter (Figure 3B). Second, FXR activation by GW4064 or CDCA increased GLP‐1R mRNA (Figure 1A,E) and protein expression (Figure 1B,F). Third, FXR KD resulted in decreased expression of GLP‐1R in INS‐1 832/13 β‐cells (Figure 2A,B). Fourth, GLP‐1R expression was substantially decreased in islets from FXR KO mice (Figure 2C,D). Fifth, GW4064 potently enhanced insulinotropic potency of GLP‐1 in control mice, whereas failed to do so in GLP‐1R KO mice (Figure 6E). It is also important to note that FXR activation led to increase in recruitment of the epigenetic regulator SRC1 to the *Glp‐1r* locus (Figure 4C). SRC1‐mediated ACH3 can potently promote gene transcription, since acetylation of nucleosomal histones promotes transcription at the target DNA locus.^28^ In agreement, loss of GW4064‐enhanced GLP‐1R expression in shSRC1 INS‐1 832/13 cells confirms that SRC1 is responsible for FXR‐regulated GLP‐1R expression.

Our data showed that the islets from diabetic GK rats displayed a substantial decreased expression of GLP‐1R (Figure 5A,B). This is likely to impair the ability of GLP‐1 to enhance cAMP generation and GSIS (Figure 5C,D). This is indeed in accordance with diminished insulinotropic potency of GLP‐1 and incretin defect observed in T2D.^14^, ^31^, ^32^ It is also necessary to note that, although chronic hyperglycaemia decreases GLP‐1R expression, it does not affect expression of PKA and Epac2 in β‐cells.^15^ Thus, decreased incretin potency of GLP‐1 would be attributed to decreased expression of GLP‐1R, rather than alterations of the major downstream effectors of cAMP in diabetic β‐cells. Importantly, our data further showed that RYGB restored expression of GLP‐1R in islets from diabetic GK rats, an effect dependent on FXR, since ablation of *FXR* completely abolished the effect of RYGB on GLP‐1R expression (Figure 6C,D). These findings are important since BAs and its nuclear receptor FXR play a critical role in improved glucose homeostasis and remission of diabetes after RYGB.^17^, ^18^

RYGB procedure results in accelerated transit of nutrients, which enhances post‐prandial GLP‐1 release from L‐cells in the ileum and colon.^6^, ^30^ It is therefore suggested that increased circulating level of GLP‐1 could be a contributor to enhanced incretin effect after RYGB.^4^ However, it is important to note that GLP‐1 is rapidly cleaved and inactivated by the ubiquitous enzyme DPP‐4, which results in a plasma half‐life of 1‐2 minutes in mammals.^33^, ^34^ These findings argue that the durable enhanced incretin effect cannot simply be attributed to increased level of the incretin hormone after RYGB. Given the critical role of GLP‐1R in the beneficial effects of RYGB on glycemic control^35^ and coupled with the new findings in this study, we can conclude that FXR‐mediated expression of GLP‐1R plays an important role in enhanced incretin effects in diabetes after RYGB. In addition to GLP‐1, the incretin hormone glucose‐dependent insulinotropic polypeptide (GIP) is also the contributor to incretin effect. However, unlike the situation in enhancing GLP‐1R expression, RYGB does not affect expression of GIP‐R in β‐cells.^16^ Thus, RYGB‐enhanced incretin effect would be largely attributed to FXR‐regulated expression of GLP‐1R in β‐cells.

Overall, our results in the current study unravel the previously unappreciated link between RYGB and BAs/FXR/SRC1 axis‐mediated epigenetic regulation of GLP‐1R expression in β‐cells (Figure 6F). Apparently, signals strongly stimulating FXR would promote expression of GLP‐1R. This is interesting given the GLP‐1R‐based therapies have become an established option for the long‐term preservation of β‐cell function.^36^ Further exploration of FXR/SRC1‐GLP‐1R signalling pathway may help to define novel approaches to treatment of T2D.

## CONFLICT OF INTEREST

The authors confirm that there are no conflicts of interest.

## AUTHOR CONTRIBUTIONS

**Xiangchen Kong:** Data curation (lead); Funding acquisition (supporting); Investigation (lead). **Linxian Feng:** Investigation (supporting). **Dan Yan:** Investigation (supporting). **Bingfeng Li:** Investigation (supporting). **Yanhui Yang:** Investigation (supporting). **Xiaosong Ma:** Funding acquisition (lead); Project administration (lead); Supervision (lead); Writing‐original draft (lead); Writing‐review & editing (lead).

## Supporting information

Supplementary Material

## Data Availability

All data sets used and/or analysed in the present study are available from the corresponding author upon reasonable request.
