# Supplementary material for: FXR‐mediated epigenetic regulation of GLP‐1R expression contributes to enhanced incretin effect in diabetes after RYGB
Source: J Cell Mol Med. 2021 Feb 21;28(6):e16339. doi: 10.1111/jcmm.16339 (PMC10941525; doi:10.1111/jcmm.16339)
Supplement: Supplementary file 1 — Supplementary Material [file JCMM-28-e16339-s001.docx]

**SUPPLEMENTARY MATERIALS**

**Table 1. List of primers used for real-time PCR**

| Gene | Primers |  |
| --- | --- | --- |
| *Glp1r* | Forward 5’-ATCGCTTCAGCCATCCTT-3’  Reverse 5’-ATACACGCCTTCCACCAG-3’ | |
| *Gapdh* | Forward 5'-CCTTCATTGACCTCAACTAC-3'  Reverse 5'-TCGCTCCTGGAAGATGGTGAT-3' | |
| *Actin*  (beta) | Forward 5'-GTAAAGACCTCTATGCCAACA-3'  Reverse 5'-GGACTCATCGTACTCCTGCT-3' | |

**Table 2. List of primers used for *Glp-1r* promoter PCR**

| Gene | Primers |
| --- | --- |
| *Glp1r* promoter | Forward 5’-GGGGTACCGCCACTACAGTTCTTCATCTT-3’  Reverse 5’-CCCAAGCTTCTCAGGACTGGGTCATAGC-3’ |
| *Glp1r* promoter  mutation | Forward 5’-GAGGCCAGTGAAGGTGTAGGATTGGTACAGTTCAGCAGTGTTTGTCAGAC  TTCTGGAAAGTATC-3’  Reverse 5’-GATACTTTCCAGAAGTCTGACAAACACTGCTGAACTGTACCAATCCTACA  CCTTCACTGGCCTC-3’ |

**Table 3. List of primers used for *Glp-1r* ChIP assay**

| Gene | Primers |  |
| --- | --- | --- |

| *Glp1r* promoter | Forward 5’-CCCTGAGGACTCTTCTGC-3’’  Reverse 5’-CTCAACTAAGGAAAGCCAC-3’ |
| --- | --- |
